# Varicocele: To Treat or Not to Treat?

**DOI:** 10.3390/jcm12124062

**Published:** 2023-06-15

**Authors:** Antonio Franco, Flavia Proietti, Veronica Palombi, Gabriele Savarese, Michele Guidotti, Costantino Leonardo, Fabio Ferro, Claudio Manna, Giorgio Franco

**Affiliations:** 1 Department of Urology, Sant’Andrea Hospital, “Sapienza” University of Rome, 00185 Rome, Italy; antonio.franco@uniroma1.it; 2 Department of Maternal and Child Health and Urological Sciences, Policlinico Umberto I, “Sapienza” University of Rome, 00185 Rome, Italy; veronica.palmbi@uniroma1.it (V.P.); gabriele.savarese@uniroma1.it (G.S.); costantino.leonardo@uniroma1.it (C.L.); giorgio.franco@uniroma1.it (G.F.); 3 Department of Urology, Nuovo Ospedale dei Castelli, 00040 Rome, Italy; michele.guidotti@gmail.com; 4 Mater Dei Hospital, 00197 Rome, Italy; fabioferro.andrologia@gmail.com; 5 Biofertility IVF and Infertility Center, 00198 Rome, Italy

**Keywords:** varicocele repair, varicocelectomy, ART, infertility

## Abstract

Varicocele treatment in infertility still remains controversial. It is clear, in fact, that in many patients, varicocele has no impact on fertility. Recent scientific evidence demonstrated that varicocele treatment is beneficial in improving semen parameters and pregnancy rate when an appropriate selection of patients is made. The purpose of treating varicocele in adults is mainly to improve current fertility status. On the other hand, the goal of treatment in adolescents is to prevent testicular injury and maintain testicular function for future fertility. Hence, the key to the success of varicocele treatment seems to be a correct indication. The aim of this study is to review and summarize current evidence in managing varicocele treatment focusing on the controversies regarding surgical indications in adolescent and adult patients, and in other specific situations such as azoospermia, bilateral or subclinical varicocele, and prior to ART.

## 1. Introduction

A varicocele is defined as an abnormal dilatation and/or tortuosity of the pampiniform venous plexus in the scrotum. It is a pathological condition caused by an alteration in the drainage of the testicle due to venous reflux in the internal spermatic vein (ISV). In fact, the left side is mainly affected due to anatomical reasons related to the ISV. In a previous study, using femoral and spermatic venographies, we observed the exclusive involvement of ISV in primary and recurrent varicoceles [1].

The condition occurs in 15% of the healthy general male population, in 35% of men with primary infertility, and in up to 80% of men with secondary infertility [2]. Several different clinical [3] and US sonographic classifications have been proposed for varicocele assessment, but unfortunately, there is no standardization, and a clear consensus has not yet been reached, which obviously also leads to difficulties in comparability [4]. According to the fourth edition of WHO classification [5], there are three grades of varicocele depending on the severity of it, from 1 to 3, with no reference to an absolute measure of the vein diameter or sonographic evidence of reflux with velocity measurement. On the other hand, Sarteschi describes a five-part classification, depending on the presence of dilated veins while supine and/or standing, the anatomical relationships of the dilated veins with the testis, the characteristics of reflux, and testicular size [6]. Cavallini et al. focused on varicocele grade and degree of reflux, showing that surgery to improve OAT and, thus, chances of successful ART should be reserved for Dubin and Amelar grade 2 and grade 3 varicoceles with continuous venous reflux at duplex Doppler assessment [7]. Furthermore, no global consensus has been established on the need for sonographic examination in the diagnosis of varicocele: on the first hand AUA/ASRM guidelines sustain that scrotal ultrasound should not be routinely performed in the initial evaluation of the infertile male; on the other hand, the EAU Guidelines, in accordance with the European Society of Urogenital Radiology Scrotal and Penile Imaging Working Group, consider scrotal Doppler necessary if physical examination is inconclusive or semen analysis remains unsatisfactory after varicocele repair to identify persistent and recurrent varicocele [8,9,10].

Varicocele may cause spermatogenetic damage resulting in altered seminal parameters, abnormalities in the development and growth of the affected testis, and, rarely, symptoms such as discomfort and pain [11]. Thus observational studies suggest that men with a varicocele tend to have a higher proportion of spermatozoa with fragmented DNA, lower total sperm counts, lower progressive sperm motility, lower sperm vitality, and higher abnormal forms when compared to control groups [12]. The exact pathophysiology and, especially, the cause–effect relationship between the presence of varicocele and abnormalities of the semen analysis has not been clearly established [13]. Conversely, a recent systematic review and meta-analysis provide a high level of evidence in favor of a positive effect of VR to improve conventional semen parameters in infertile men with clinical varicocele [14].

The aim of our study is to review the latest reports on varicocele treatment and provide simple and practical steps for managing a correct indication of treatment, focusing on the controversies on this issue.

## 2. Surgical Treatment

Several therapeutic options are available for varicocele treatment and may involve an endovascular or surgical approach [13].

In Europe, endovascular techniques are popular due to their minimally invasive nature, despite their higher recurrence rates [15]. They include:

1. Retrograde sclero-embolization, trans-femoral or trans-brachial;
2. Antegrade sclerotherapy (Tauber technique) [16].

Concerning surgical approach, this may be:

- Retroperitoneal;
- Inguinal;
- Sub-inguinal.

The retroperitoneal access involves an incision in the supra inguinal site and the ligation of the ISV immediately above the internal inguinal ring (Ivanissevich technique) or higher up at the level of the anterior iliac spine (Palomo technique).

**Figure 1:**
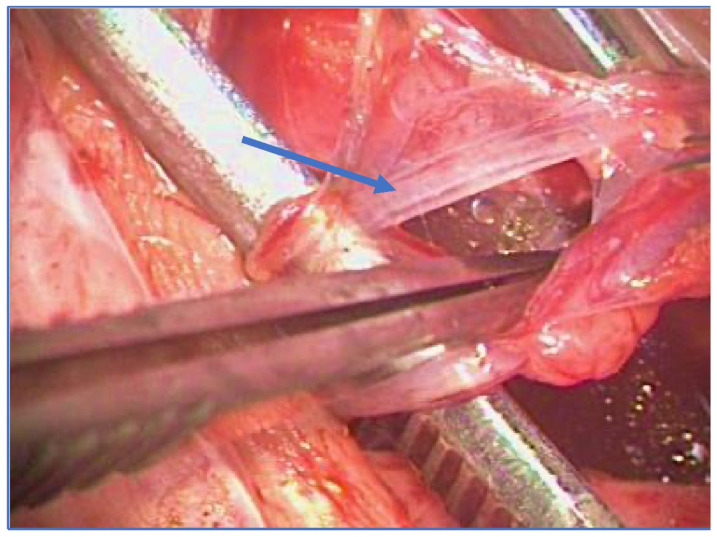
Microsurgical lymphatic sparing Palomo technique (arrow: spared lymphatics).

A recent Palomo technique variant consists of sparing a few lymphatic vessels with the aid of an operating microscope, to avoid post-operative hydrocele which might occur after the standard Palomo procedure [17,18,19,20]. The approach is moved from the sub-inguinal to the pre-peritoneal level, just above the internal inguinal ring, as described by Jones for nonpalpable testes [21]. The internal spermatic veins are reached by splitting the muscle plane and thus preserving the integrity of the inguinal channel. The deferential vein can be evaluated but not necessarily ligated, considering that dilation is not synonymous with reflux [17] (Figure 1).

Retroperitoneal repair can also be performed using the laparoscopic technique, particularly in bilateral disease [22]. In our view, laparoscopic repair appears to be more invasive and costly than the other techniques, due to general anesthesia, pneumoperitoneum issues and extremely rare but possible dreadful complications [23]; however, it has the advantage of excellent visibility of the posterior abdominal wall allowing a thorough search of sites known to be responsible for recurrent varicoceles, such as renal, caval and pelvic cross-over veins. Moreover, optical magnification optimizes the surgeon’s ability to preserve the testicular artery and lymphatic channels while ligating all veins to minimize the risk of hydrocele formation or varicocele recurrence. Finally, the laparoscopic approach allows a simultaneous correction of bilateral varicocele. Some evidence demonstrates lower recurrence rates (3–6%), especially when compared to sclero-embolization procedures, which were shown to reach 4–11% of varicocele recurrence [24]; furthermore, sclero-embolization procedures have their specific complications, such as inadvertent femoral artery perforation, radiation exposure, sclerosant agent local irritation or orchitis and coil migration that, despite being very uncommon, should be taken into account [25,26].

The sub-inguinal microsurgical technique, instead, is widely used in the United States with very low reported recurrence rates [27,28].

Different subinguinal microsurgical modified approaches have been subsequently proposed [27,29]. According to EAU guidelines and AUA/ASRM [30], the use of an operating microscope makes microsurgical subinguinal varicocelectomy the preferred method of treatment due to its lower incidence of complications and recurrence rates, as well as its potential for greater improvement in semen parameters [30,31]. However, it is technically demanding and needs access to an operating microscope [32].

Regarding the outcomes of the different techniques, there is still debate in the literature: a meta-analysis by Cayan et al. suggested that surgical intervention was better than embolization with regard to spontaneous pregnancy rates (41.97% for microsurgery versus 33.20% for embolization, *p* = 0.001) [23] and recurrence rates (1.05% for microsurgery versus 12.70% for embolization, *p* = 0.001) [23]. A randomized controlled trial (RCT) from Al-Kandari et al. demonstrated that, compared with open inguinal and laparoscopic varicocelectomy, subinguinal microsurgical varicocelectomy offers the best outcome only in terms of complications (hydrocele) and recurrence [33]. In our experience, the modified lymphatic-sparing Palomo technique appears to offer excellent results in terms of outcomes and complication rates: one of the authors (F.F.), in the period January 2009–September 2020, treated 633 children and adolescents using this technique with a recurrence rate of 2.8% and postoperative hydrocele rate of 0.4% (unpublished data). Therefore, no technique has been proven to be certainly superior to the others. Each one of them has its own advantages and disadvantages (Table 1). High-qualities studies comparing different surgical approaches are still missing, and results of the available studies in the literature appear to be inconclusive.

## 3. Varicocelectomy Indications

The role of varicocele treatment in improving sperm parameters and, most importantly, pregnancy and live birth rate, in other words, in enhancing male fertility, has represented a matter of discussion between authors and specialists.

While initially, some studies and metanalysis denied benefits on fertility in patients treated for varicocele [34,35], recent scientific evidence demonstrated that varicocele treatment improves semen parameters and pregnancy rates, when an appropriate selection of patients is made [22,36,37].

A recent global survey involving 574 experts from 59 countries showed wide disagreement regarding varicocele management and poor adherence to guidelines [37]. Current American Urological Association (AUA) and European Urology Association (EAU) guidelines suggest treating varicocele in well-selected patients when specific conditions are present [9,28].

We will focus on the main indications for varicocelectomy, seeking to answer the relevant question of whether or not varicocelectomy improves the fertility status of the patient. Various clinical scenarios involving varicocele repair (VR) will be discussed below in detail:

- VR in children and adolescents;
- VR in infertile couples, oligoasthenoteratozoospermia and sperm DNA fragmentation;
- VR in azoospermia;
- VR prior to assisted reproductive techniques (ART);
- VR in subclinical varicocele;
- VR in bilateral varicocele.

### 3.1. Role of Varicocelectomy in Children and Adolescents

A particular situation is represented by the presence of varicocele in children and adolescents. In this delicate period of life, the presence of a varicocele might jeopardize normal testicular growth and impair the spermatogenetic process, and it would be reasonable to believe that the early repair of this vascular anomaly might prevent testicular damage [38]. However, there is still confusion about who should be treated in the pediatric population. Without the aid of a semen analysis, the selection of the children to be treated is only based on the presence of reduced growth of the affected testis (testicular hypotrophy). Instead, in adolescents, current recommendations for VR are based on the clinical findings of impaired testicular growth and/or altered seminal parameters when available [39].

Important studies have focused on this issue; in particular, Cayan et al. evaluated 408 patients (age 12–19) with clinical varicocele undergoing microsurgical varicocelectomy vs. observation only. Their results showed a significant increase in paternity rates, reduced time to conception and no additional treatment necessary to conceive post-operatively in adolescents who received VR compared to ones observed only. In particular, patients with varicocele who underwent microsurgical VR will have better sperm parameters and 3.63 times increased odds of achieving paternity compared to controls not undergoing varicocele surgery and followed conservatively [40].

An interesting meta-analysis by Silay et al. on the treatment of adolescent varicocele states that “moderate evidence exists on the benefits of varicocele treatment in children and adolescents in terms of testicular volume and sperm concentration. Current evidence does not demonstrate the superiority of any of the surgical/interventional techniques regarding treatment success. Long-term outcomes including paternity and fertility still remain unknown” [41]. The editorial of this article, by J. Elder, concludes that RCTs will be necessary to “*prevent a potentially damaging process from going untreated, while at the same time avoiding unnecessary interventions for a highly prevalent condition*” [42].

Current European Association of Urology (EAU) guidelines recommend treating varicocele when one of the following conditions is present: (A) is associated with testicular hypotrophy (size difference >20%), (B) an additional testicular condition affecting fertility is present, (C) is symptomatic and (D) a pathological sperm quality is detected. According to a recent study, varicocelectomy in adolescents may also be associated with increased sperm DNA integrity and mitochondrial activity [43]. Based on current evidence, all these indications are discussed in detail in a recent review by Cannarella et al. who created a flow chart for the management of childhood and adolescent varicocele: conservative management may be suggested in patients with peak retrograde flow (PRF) <30 cm/s, testicular asymmetry <10% and no evidence of sperm and hormonal abnormalities; in patients with 10–20% testicular volume asymmetry or 30 < PRF ≤ 38 cm/s or sperm abnormalities, careful follow-up may ensue. In the case of absent catch-up growth or sperm recovery, varicocele repair should be suggested. Finally, treatment can be proposed at the initial consultation in painful varicocele, testicular volume asymmetry ≥20%, PRF > 38 cm/s, infertility and failure of testicular development [44].

### 3.2. Role of Varicocelectomy in Infertile Couples, OAT and DNA Fragmentation

Since the Evers and Collins meta-analysis, stating that varicocele treatment had no role in improving couple infertility, a large mass of new studies, including RCTs, global consensus surveys and meta-analyses, have been published, which also demonstrated a significant role of treatment in improving sperm parameters and pregnancy rates [36,37,45,46]. The same Evers and Collins group in 2012 published a new meta-analysis, which concluded that the treatment of varicocele in men from couples with otherwise unexplained subfertility may improve a couple’s chance of pregnancy [22]. A meta-analysis from Marmar et al. supporting this hypothesis reported a pregnancy rate of 33% (31 of 96) in surgically treated men compared with 15.5% (27 of 174) in untreated men, corresponding to an OR of 2.87 (95% CI 1.33–6.20). The analysis included two randomized trials and three observational studies comprehending infertile men with an abnormal semen analysis and a palpable varicocele [47]. These data are in line with those obtained in another randomized controlled trial by Abdel-Meguid and colleagues, in which a similar odds ratio for achieving a spontaneous pregnancy after varicocelectomy was reported (OR 3.04; 95% CI 1.33–6.95) [36].

Overall, the available evidence supports a beneficial effect of varicocelectomy on pregnancy outcomes. In fact, the Cochrane reviews denying the beneficial role of VR have been criticized for their inclusion of men with subclinical varicocele and normal semen parameters [22]. Therefore, sufficiently powered RCTs with homogenous patient populations are needed to overcome these partly conflicting results.

The key point, in our view, is the correct indication to treat varicocele, and selecting the right patients to treat will lead to a significant improvement in their fertility.

Both EAU and AUA guidelines suggest the treatment of varicocele in infertile couples [8,9]. However, as far as infertile couples are concerned, EAU guidelines discourage treatment in men who have normal semen analysis and/or subclinical varicocele (grade A recommendation) and suggest treatment in those with clinical varicocele, oligospermia and otherwise unexplained infertility in the couple (grade A recommendation).

Varicocelectomy represents a useful and generally simple procedure for the treatment of men with oligoasthenoteratozoospermia (OAT), and this is often expressed in global practice patterns and in the EAU and AUA/ASRM guidelines. In fact, they recommend surgical treatment if a palpable varicocele and infertility are associated with “abnormal semen parameters, except for azoospermic men”. The latest Cochrane review about this issue suggests an improvement in pregnancy rates for men with OAT who underwent VR, but it is uncertain whether live birth rates increase as well [48].

Sperm DNA fragmentation (SDF) has emerged as an important measure of sperm function and a predictor of reproductive outcomes. VR is associated with an improvement in SDF, including both single-strand and double-strand DNA fragmentation, as well as seminal oxidative stress [49,50,51]. Two recent meta-analyses calculated a mean reduction in SDF after VR of 7.23% and 6.14%, respectively [50,52]. According to a recent study from Yan et al., the possible role of varicocele treatment in improving sperm DNA fragmentation in infertile couples should urge a change in the current guidelines on varicocele treatment [53]. Concerning the guidelines, an important discrepancy between their statements and current evidence must be acknowledged. In fact, AUA/ASRM declare “*there are no well-controlled studies that VR will reduce risk of recurrent pregnancy loss in men with elevated SDF*”. On the other hand, several studies have confirmed the role of varicocelectomy in improving semen quality, increasing the pregnancy rate, and significantly decreasing the miscarriage rate [54,55]. To note, Ghanaie et al. evaluate the effects of varicocelectomy on semen parameters, pregnancy rates, and live birth in couples with first-term recurrent miscarriage in a randomized-control trial and their results showed a significant difference in the varicocele repair arm, in terms of improved outcomes [56]. EAU guidelines report that there is “increasing evidence” that VR may improve SDF and ART outcomes and recommends VR for men with raised SDF and failed ART. Finally, a recent global survey on the management of SDF states that there are no specific recommendations regarding the general approach to managing infertile men with elevated SDF in the guidelines; however, possible first-line treatments consist of lifestyle modification strategies, including maintaining a healthy lifestyle to overcome obesity, the cessation of smoking and alcohol use, as well as treating genital infections and eliminating toxic exposure [57].

Concerning the male age factor, controversy exists as to whether varicocelectomy is as effective in older men, as it is believed that long-standing varicoceles can cause irreversible testicular damage, or that older testes may have limited potential for recovery from varicocele-induced damage [58]. The clinical implication is that if varicocelectomy is less effective in older men, perhaps it should not be offered, with men electing assisted reproduction instead. However, some studies [59,60] showed that age does not necessarily need to be an exclusion factor for varicocele treatment. In fact, evaluating varicocele’s outcomes in couples of different ages, Firat and Erdemir [59] found increased semen parameters; although pregnancy rates after varicocelectomy were higher in the younger group compared with the others, this difference was not statistically significant. Therefore, even couples with male partners over 35 years of age might have a reasonable chance of natural pregnancy after VR. Naturally, the female age factor tends to be more important in an infertile couple, and paternal age contributes relatively smaller to the overall age-related decline in the fertility of a couple when compared with maternal age.

**Figure 2:**
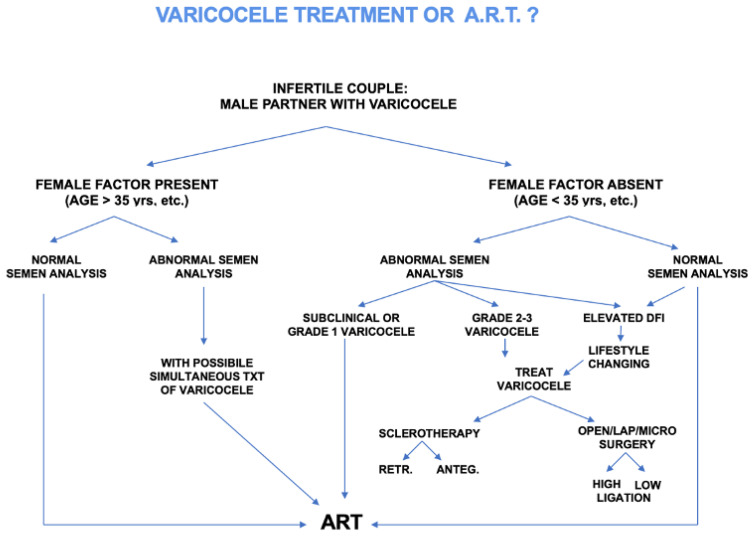
Flow chart analyzing indications to treat varicocele or perform ART.

In the conclusion of this chapter, very often, the urologist is faced with the dilemma of treating varicocele or sending the couple directly to ART. A flowchart for the treatment of varicocele or ART in infertile couples is presented in Figure 2.

### 3.3. Role of Varicocelectomy in Azoospermia

As stated recently in a global consensus on the management of varicocele for male infertility, there is a wide discrepancy in dealing with an azoospermic patient affected by varicocele [37]. Starting from the evidence supported by AUA/ASRM, the guidelines state that “the couple should be informed of the absence of definitive evidence supporting VR prior to ART”. The EAU guidelines instead declare that VR in men with NOA may result in the appearance of sperm in the ejaculate (20.8% to 55%) and is associated with improved surgical sperm retrieval rates (OR, 2.65; 95% CI, 1.69–4.14). However, it cautions that the evidence is based on observational studies only and suggests fully discussing the risks and benefits of VR with the patient with NOA and a clinical varicocele. Certainly, clinicians must first evaluate medical history, genetic testing, and hormonal exams to distinguish obstructive azoospermia (OA) from non-obstructive azoospermia (NOA). Then, it is necessary to exclude the possibility that the varicocele is an incidental finding in a patient with azoospermia certainly unrelated to varicocele. If such conditions are excluded, a varicocelectomy may be performed in men with NOA, resulting in beneficial effects on sperm retrieval rates (SRR), as demonstrated in a recent meta-analysis showing an increased SRR in men with NOA who underwent varicocelectomy compared with men with NOA who did not undergo varicocelectomy (OR 2.65; 95% CI 1.69–4.14; *p* < 0.001) [61]. Despite these results, there is still reluctance in offering VR to these patients: a major criticism is that in almost all cases published, the sperm count achieved in the ejaculate is very low, and ICSI is still needed [62]. Sometimes, the appearance of sperm is only transitory [63,64]. Furthermore, none of these studies are controlled, and the appearance of sperm in these men may be due to spontaneous variation and not be due to the VR [9]. A study observed a beneficial effect of VR only in azoospermic patients with a testicular histologic pattern of hypospermatogenesis or late maturation arrest (MA) while those with Sertoli cell-only syndrome (SCOS) showed no change [65]. However, scheduling a testicular biopsy routinely prior to VR in azoospermic patients might be difficult to accept.

In conclusion, we believe that the selection of NOA patients for varicocele repair remains a matter of personal belief and choice. In our view, the advantages of VR in azoospermic patients are very limited and rarely of clinical significance.

### 3.4. Role of Varicocelectomy Prior to ART

Correcting a varicocele before proceeding with IVF-ICSI is a controversial topic and many ART centers do not even consider the presence of a clinical varicocele.

As previously mentioned, there is fair evidence that the surgical repair of clinical varicocele may improve semen parameters and may decrease seminal oxidative stress and sperm DNA fragmentation, thus increasing the chances of natural conception. However, it is unclear whether performing varicocelectomy in men with clinical varicocele prior to ART may improve treatment outcomes [66].

Support for VR before ART is derived from the fact that surgical VR is certainly a minor and less expensive procedure than ART itself. Furthermore, VR might improve semen quality and facilitate spontaneous pregnancies or enhance the success rate of ART. Positive outcomes are illustrated in a meta-analysis by Esteves et al. who reported increased clinical pregnancies (OR = 1.59, 95% CI: 1.19–2.12, I^2^ = 25%) and birth rate (OR = 2.17, 95% CI: 1.55–3.06, I^2^ = 0%) in patients who underwent varicocelectomy prior to ART vs. ART without VR [67]. Another meta-analysis by Kirby et al. found that VR improved the ART live birth rate in men with oligospermia (odds ratio [OR], 1.699) [68]. On the other hand, VR might delay the ART procedure by 6 to 12 months for an uncertain benefit; the presence of female factors (age >35 years, etc.) may induce clinicians to immediately offer ART-avoiding VR. However, our policy in the case of advanced female age and indications of varicocele correction is to offer immediate ART together with VR. In this way, there is no delay in ART, but in the event of an unsuccessful result of it, the couple will benefit from the advantages of VR. It is clear, hence, that the decision to perform VR before ART should be individualized based on other variables such as the female partner’s age, history of prior failure, varicocele grade, SDF levels, duration of infertility, etc., and, of course, wide counseling of the infertile couple. As a matter of fact, different studies have investigated several factors implicated in ART success rate after VR [69,70]. EAU guidelines do not indicate whether VR prior to IVF will improve pregnancy rates but suggest VR in men with OAT or when elevated SDF is present [9].

Another aspect that may be considered is the cost-effectiveness of the procedures involved in ART. The cost of the various ART procedures is an important consideration for couples and society, considering that often coverage for these procedures is not provided routinely and there is wide variability of cost-effectiveness when comparing across various ART procedures [71]. For instance, Dubin et al. recently demonstrated through a cost-effectiveness analysis that varicocelectomy increases semen parameters in severely oligospermic patients, thus providing previously ineligible couples an opportunity to elect for intra-uterine insemination (IUI), a less invasive and less expensive alternative to in vitro fertilization (IVF) or intracytoplasmic sperm injection (ICSI) [72].

### 3.5. Role of Varicocelectomy in Subclinical Varicocele

Another complex topic about the indication of varicocelectomy involves subclinical varicocele. In fact, considerable confusion and diversity of opinion and practice appear to be when it comes to subclinical and grade 1 clinical varicoceles too.

On the one hand, clinicians often do not believe that correcting a grade 1 varicocele is of benefit, and, usually, its repair is not recommended. On the other hand, paradoxically, when there is nothing to offer to a man with idiopathic OAT, for instance, many clinicians would recommend VR if a varicocele was detected through US [37].

Again, even though guidelines are clear in recommending against subclinical varicocele repair, there are nevertheless studies claiming some benefit from varicocelectomy in this type of patient [73,74]. This statement is confirmed by evidence from the literature which analyzed fertility and semen parameters outcomes in subclinical varicocele repair. In a randomized controlled trial by Yamamoto et al., men with subclinical varicocele received either high ligation or no treatment. No difference in terms of pregnancy rates was found (6.7% versus 10%, *p* = 0.578), although those who underwent high ligation demonstrated significant increases in sperm density and total motile sperm (*p* < 0.006 and *p* < 0.008, respectively) [75].

Grasso et al. investigated 68 men with a left-sided subclinical varicocele who randomly underwent either high ligation or no treatment and showed no improvement in semen quality or pregnancy outcomes in either group [76].

Notwithstanding the heterogeneity of these studies, depending on different diagnostic methods, different surgical techniques and different patients’ characteristics, these biases highlight the lack of standardization, which makes drawing comparisons difficult. In our opinion, subclinical varicocele seems to be a para-physiological condition and there is no evidence of the efficacy of its treatment on improving semen parameters and pregnancy outcomes; thus, varicocelectomy should only be offered to men presenting with clinically palpable varicoceles, preferably grade 2 or 3.

### 3.6. Role of Varicocelectomy in Bilateral Varicocele

The bilateral ligation of the spermatic veins has also been debated between urologists and andrologists. Regarding the anatomy, the left gonadal vein drains perpendicularly into the left renal vein, and the “nutcracker” effect on the left renal vein of the compass between the aorta and the superior mesenteric artery results in higher hydrostatic pressure in the left renal vein with increased chances of venous reflux into the left internal spermatic vein when compared to the right one, which drains directly into the inferior vena cava. The study of Pallwein et al. [77] confirms this phenomenon, showing a significantly higher varicocele recurrence rate in patients with left renal vein entrapment compared with patients without. Therefore, venous reflux on the right side seems to be very unlikely. However, particular relevance should be placed on the rare true cases of bilateral clinical varicocele or on the more frequent cases of a left-sided grade 2 or 3 clinical varicocele combined with a subclinical or grade 1 right-sided varicocele [78]. More specifically, an extremely rare case report of isolated right-sided varicocele diagnosed after an extensive work-up was reported in a patient with venous anomalies and a spontaneous portosystemic shunt [79].

Once again, European and American guidelines are not clear on whether or not to treat a subclinical right-side varicocele in the presence of a left-side clinical varicocele.

Recent studies suggested that bilateral varicocelectomy is better than unilateral to improve spontaneous pregnancy rates in patients with left clinical and right subclinical varicocele [80,81,82]. Among these, a meta-analysis of four RCTs reported no significant difference in sperm concentration and motility between the two groups, but the spontaneous pregnancy rate showed an odds ratio of 1.73, suggesting better results in the bilateral ligation group [82]. Another prospective randomized trial from Sun et al. demonstrated the same results as the previous study, confirming the role of bilateral varicocele treatment [83]. Indeed, there are some limitations upon those trials: only spontaneous pregnancy was evaluated, rather than assisted reproductive pregnancy, and thus it may affect conclusions. Furthermore, as stated by the authors, different surgeons, different surgical techniques and different follow-up times may have led to different rates of spontaneous pregnancy rates.

With this knowledge in mind, a definitive recommendation cannot be made. In our opinion, the rare true clinical bilateral varicocele deserves bilateral treatment, while the more frequent grade 1 or subclinical reflux on the right side accompanying grade 2 or 3 varicocele on the left one should receive repair only on the left side.

**Table 1:** Varicocele surgical techniques: pros and cons.

Technique	Pros	Cons
Open retroperitoneal high ligation (Palomo) [23,33,48,83]	Complete ligation	General anesthesia, Higher hydrocele risk
Microsurgical lymphatic sparing Palomo [13,17,18,19,20]	Complete ligation Lower hydrocele risk	General anesthesia Access to operating microscope
Microsurgical subinguinal or inguinal surgery [23,28,33]	Less invasive (local anesthesia) Lower recurrence rate Lower hydrocele risk	Access to operating microscope Longer surgical time
Laparoscopic surgery [23,32,33]	Bilateral varicocele Higher magnification Lower recurrence rate	High costs More invasive (intraperitoneal) General anesthesia
Sclero-embolization [15,16,23,24,25,26,48]	Minimally invasive Short time Outpatient	Limited applicability Higher recurrence rate Radiation exposure

## 4. Summary

In summary, the strongest recommendations for varicocele repair are represented by couple infertility, OAT, grade 2 or 3 clinical varicocele, partner <37 yrs, patient age <40 yrs and testicular hypotrophy in children and adolescents. Indications are reinforced when OAT is severe and in younger patients. On the other hand, little indication exists to treat varicocele in azoospermic patients. Finally, an additional indication is represented by elevated sperm DNA fragmentation, particularly in partners of women who had undergone an unsuccessful ICSI or repeated miscarriages.

## 5. Conclusions

A conclusive answer to the Hamletic doubt of the title, to treat or not to treat varicocele, is not yet possible. Varicocele is the most common correctable cause of male infertility. In selected cases, varicocele treatment is beneficial in improving semen parameters and pregnancy rates. On the other hand, the high prevalence of the disease, together with the knowledge that many patients with varicocele are fertile, might lead to overtreatment. Urologists and andrologists, as well as any clinician who plays an important role in dealing with this common disease, must carefully counsel patients after having analyzed their history, physical examination findings and all pertinent clinical parameters before leading them to the operating theatre.

## Data Availability

Not applicable.
